# KCND2: A prognostic biomarker and regulator of immune function in gastric cancer

**DOI:** 10.1002/cam4.6236

**Published:** 2023-06-22

**Authors:** Hongying Zhou, Dan Su, Yun Chen, Yiwen Zhang, Ping Huang

**Affiliations:** ^1^ SuZhou Medical College of Soochow University Suzhou Jiangsu Province China; ^2^ Department of Medical Oncology, Cancer Center Zhejiang Provincial People's Hospital (Affiliated People's Hospital, Hangzhou Medical College) Hangzhou Zhejiang China; ^3^ Department of Clinical Medicine Hangzhou Medical College Hangzhou Zhejiang China; ^4^ Department of Pharmacy, Center for Clinical Pharmacy, Cancer Center Zhejiang Provincial People's Hospital (Affiliated People's Hospital, Hangzhou Medical College) Hangzhou Zhejiang China

**Keywords:** gastric cancer, KCND2, M2 macrophages, NF‐κB, prognostic biomarker

## Abstract

**Background:**

Gastric cancer is a highly heterogeneous disease, which makes it challenging to develop effective targeted therapies. Although the potassium voltage‐gated channel subfamily D (KCND) channels, particularly KCND2 (also known as Kv4.2), have found evidence of involvement in the occurrence and development of various cancers, there are still some limitations in our understanding of KCND2's roles in gastric cancer.

**Methods:**

We analyzed the correlation between KCND2 expression and clinical features as well as immune infiltration using the Cancer Genome Atlas (TCGA) database. Functional assays of KCND2 were conducted using Cell counting Kit‐8 (CCK8), clone formation assay and cell cycle analysis. Additionally, immunofluorescence, flow cytometry and quantitative real‐time polymerase chain reaction (qRT‐PCR) techniques were used to investigate tumor proliferation and immune cell infiltration at different levels of KCND2 expression in vivo.

**Results:**

KCND2 was markedly elevated in gastric cancer and its expression appeared to link to different grades, T stages, and N stages. In addition, KCND2 was an independent predictor of prognosis, and its elevated levels in TCGA database revealed a more unfavorable prognosis for patients with gastric cancer. KCND2 strengthened the viability at the cellular level by boosting the proliferation of gastric cancer cells and reducing their death rate. Additionally, it also highlights that KCND2 the abilities of proliferating of gastric cancer cells by stimulating NF‐κB both in cell and animal levels. In addition, the findings provided proof that in animal levels, KCND2 might regulate the immune system by associating with promoting M2 macrophages, which are known to play critical roles in cancer progression. Mechanistically, KCND2 was found to lead to the infiltration of M2 macrophages through activation of NF‐κB, ultimately promoting the advancement of gastric cancer.

**Conclusion:**

Overall, these findings suggest that KCND2 is likely to be available as an underlying therapeutic target for gastric cancer.

## 1 INTRODUCTION

Gastric cancer is considered one of the predominant leading causes of mortality all over the world.^1^ It is a highly molecularly and phenotypically heterogeneous disease.^2^ Although early gastric cancer has an almost over 90% survival rate (5‐year survival rate), unfortunately the diagnosis rate is extremely low and a large majority of patients are already diagnosed with advanced gastric cancer.^3^ Advanced gastric cancer is mainly treated with sequential chemotherapy regimens, with platinum and fluoropyrimidine‐based chemotherapeutic agents; however, the median survival is usually <1 year.^1^ Currently clinically approved targeted agents for gastric cancer include trastuzumab, ramucirumab, and nivolumab or pembrolizumab.^1^ Therefore, it is particularly important to find a universal molecule that can diagnose gastric cancer at an early stage.

Voltage‐gated potassium (Kv) channels are able to regulate the cell membrane potential of excitable cells, especially neurons and myocytes.^4^ Several reports have shown that Kv channels can mediate the processes of cell proliferation, cell division, and apoptosis, while its expression can also be activated in non‐excitable cells under specific conditions such as hyperpolarization.^5^, ^6^, ^7^, ^8^ Recent literature has reported that Kv channels are remodeled in many types of cancer, such as gastric, lung, and breast cancers.^9^ The potassium voltage‐gated channel subfamily D (KCND, also known as Kv4) consists of three members, including KCND1 (Kv4.1), KCND2 (Kv4.2), and KCND3 (Kv4.3), which have been widely documented to be responsible for the initiation and advancement of cancers.^10^, ^11^, ^12^, ^13^

Despite the association of KCND channels, especially KCND2, with the development and progression of various cancers, there remain some limitations in our understanding of the involvement of KCND2 in gastric cancer. For example, the mechanisms of how KCND2 promotes gastric carcinogenesis are still poorly understood. Furthermore, studies have focused on the association between KCND2 expression and the clinicopathologic features of gastric cancer patients, but relatively little attention has been paid to the functional consequences of KCND2 dysregulation in gastric cancer cells. Therefore, further studies are needed to elucidate the definitive roles of KCND2 in gastric cancer and to explore its potential as a diagnostic or therapeutic target for this disease.

## 2 MATERIALS AND METHODS

### 2.1 Bioinformatics analysis

In this study, we analyzed the RNAseq data of gastric cancer patients from the TCGA database (https://portal.gdc.cancer.gov) and obtained clinical information from UCSC XENA (https://xenabrowser.net/datapages/).^14^ The data were transformed into TPM format and log2 transformed for normal and cancer samples. To visualize KCND2 mRNA expression levels in normal and STAD clinical samples, we used the ggplot2 package in R language. We generated time‐dependent ROC curves using timeROC package. We also conducted fitted survival regressions using the survival package to study KCND2's survivals and the results were visualized with the survminer package. The Pearson's correlation test was used to identify significantly different genes for GO and KEGG analysis, which were then analyzed using the clusterProfiler R package.^15^ The ESTIMATE algorithm was utilized to obtain stromal, immune cell, ESTIMATE, and tumor purity scores for immune cell infiltration analysis.^16^ CIBERSORT was used to calculate the content of immune cells in each sample, and correlation analysis was performed using Spearman's correlation coefficient.

### 2.2 Cell culture and transfection

We used RPMI‐1640 medium (Gibco) with 10% fetal bovine serum (FBS, Hyclone) to culture human gastric mucosal epithelial cells GES‐1, while human gastric cancer cells (AGS, HGC‐27, SGC‐7901, MGC‐803) and mouse‐derived gastric cancer cell line MFC were grown in DMEM medium (Gibco) with 10% FBS. All cells were incubated at 37°C and 5% CO_2_. Lentiviruses carrying the KCND2 plasmid and KCND2‐specific shRNA were designed and generated by GenePharma Co. Ltd. Subsequently, GC cells were transfected with the lentiviruses containing either the KCND2 plasmid or shRNA. The transfected cells were then subjected to puromycin selection at a concentration of 1 μg/mL for a duration of 2 weeks.

### 2.3 Quantitative real‐time polymerase chain reaction (qRT‐PCR)

Total RNA was extracted from gastric cancer cells using the RNA Isolation Kit (NORGEN, cat no: 17270) according to the manufacturer's instructions and quantified it with a spectrophotometer. 500 ng of cDNA was synthesized from the RNA template using a reverse transcription kit (BIO‐RAD, #1708841), and PCR amplification was performed by adding Taq polymerase, cDNA template, primers for genes, and reference genes to the reaction mixture (QIAGEN, #339347). The primer sequences can be found below. KCND2 F: CCTACATGCAGAGCAAGCG, R: GTG GTTTTCTCCAGGCAGTG; human GAPDH: F: TGACTTCAACAGCGACACCCA, R: ACCCTGTTGCTGTAGCC AAA; mouse Arg1 F: CTCCAAGCCAAAGTCCTTAGAG, R: GGAGCTGTCATTAGGGACATCA; mouse Mrc1 F:CT ATGCGCTGCGTTATCACAG, R: AAAGAAAGTGACGAGGCAGAG; mouse IL‐6 F: TCTGGGAAATCGTGGAAATGAG, R: TCTCTGAAGGACTCTGGCTTTGTC; mouse IL‐10 F: CTTACTGACTGGCATGAGGATCA, R: GCAGCTCTAGGAGCATGTGG; mouse VEGFɑ F: CTGCCGTCCGATTGAGACC, R: CCCCTCCTTGTACCACTGTC; mouse GAPDH F: TACAGCAACAGGGTGGTGGAC, R: TGGGATAGGGCCTCTCTTGCT.

### 2.4 Clone formation assay

Gastric cancer cells are plated in 6‐well well plates at a density of 100 cells per well. Add 2 mL of complete medium to each well and gently shake the plate to ensure uniform distribution of cells. Place cells in a humidified incubator at 37°C and 5% CO_2_ and incubated for 14 days. The resulting colonies were fixed with 4% paraformaldehyde (PFA) for 20 min and stained with Crystal Violet solution (0.5% in 20% methanol) for 15 min before counting under the microscope.

### 2.5 Cell counting Kit‐8 (CCK‐8) assay

Gastric cancer cells are loaded in 96‐well plates at a density of 2000 cells/well. Gastric cancer cells transfected with shRNA were cultured for 5 or 7 days followed by the addition of CCK‐8 reagent (Dojindo Laboratories) to each well (ratio of 10 μL of CCK‐8 reagent to 100 μL of culture medium). After incubation in a humidified incubator at 37°C and 5% CO_2_ for 1 h, the absorbance of each well was measured at 450 nm. The absorbance is proportional to the number of viable cells.

### 2.6 Cell cycle

Gastric cancer cells were harvested after 5 or 7 days of transfection with shRNA. Washed the cells twice with ice‐cold phosphate‐buffered saline (PBS) and then fixed the cells with 70% ice‐cold ethanol by vortexing simultaneously and the cells were fixed overnight at −20°C. The fixed cells were then centrifuged at 300 g for 5 min and the supernatant was removed. The cell pellets were incubated in 1 mL PBS containing propidium iodide (PI) at room temperature in the dark for 30 min and then analyzed by flow cytometry.

### 2.7 Apoptosis flow assay

Gastric cancer cells were harvested and washed twice with ice‐cold PBS. The cells were then resuspended and incubated with annexin V (5 μL/test) and 7‐AAD (5 μL/test) (BD Pharmingen, #559763) for 15 min at room temperature, protected from light. After incubation, buffer was added and gently mixed, and the cells were subjected to flow cytometry as soon as possible.

### 2.8 Animal experiments

MFC cells (2 × 10^5^ in 50 μL PBS/per mouse) with different expression levels of KCND2 (control or KCND2 knockdown) were injected subcutaneously into the lateral abdomen of 12 female 615 mice to generate tumors. When the tumors grew to visualization, the mice were injected with PBS or lipopolysaccharides (LPS, 1.5 mg/kg; MedChemExpress, #HY‐D1056)^17^ through the peritoneal cavity. When the largest tumor in the control group reached a diameter of about 1 cm, the mice were euthanized, the tumors were collected, and the tumors were weighed and subjected to further experiments. Animal experiments were approved by the Animal Ethics Committee of Zhejiang Provincial People's Hospital (Ethics No. IACUC‐20230306‐03).

### 2.9 Immunofluorescence assay

For cells, glass chambers were treated with poly‐L‐lysine for 1 h, and then gastric cancer cells were grown into the chamber (FALCON, #354118), and cells were fixed with 4% PFA for 1 h at room temperature. For tissues, the tumor samples were embedded in paraffin and the samples were cut into 4 μm sections. The sections were dewaxed in xylene and then gradually dehydrated with gradient alcohol and excess wax was removed. Sections were subjected to antigen repair in Citrate solution (10 mM, pH 6.0) at 100°C for 20 min. Next, cells or slides were treated with 0.1% Triton X‐100 for permeabilization on ice for 5 min, followed by dressing with 5% BSA for 1 h at room temperature to reduce non‐specific staining. Cells or slides were incubated overnight at 4°C with primary antibodies (anti‐NF‐κB p65, Beyotime, #AN365, 1:200 dilution; anti‐Ki67, abcam, #ab15580l, 1:500 dilution; anti‐PCNA, Dako, #M0879, 1:500 dilution; anti‐CD206, Proteintech, #18704‐1‐AP, 1:200 dilution) followed by secondary antibodies (Alexa Fluor™ 555 anti‐rabbit IgG, ThermoFisher Scientific, #A‐31572, 1:500 dilution; Alexa Fluor™ 488 anti‐mouse IgG, ThermoFisher Scientific, #A‐11029) for 1 h at 37°C. Cell nuclei were stained with DAPI (Beyotime, #C1002).

### 2.10 Flow cytometry assay for immune cell infiltration

The tumors were removed, cut into small pieces with a scalpel, added to HBSS culture containing 1 mg/mL collagenase (Sigma) and 0.1 mg/mL DNAase (Sigma), and incubated for 1 h at 37°C for digestion. The digests were sieved with 70 μM filters and the cells were collected by centrifugation and washed with PBS containing 2% FBS to prepare a tumor single‐cell suspension. Cell suspensions were then incubated with FcRblock (1 μg/test; BioLegend, #101319) for 10 min at 4°C, followed by treatment with CD45 (PE anti‐mouse CD45, 0.5 μg/test; BioLegend, #157603), F4/80 (Alexa Fluor® 488 anti‐mouse F4/80; 1 μg/test, BioLegend, #123119), CD11b (PerCP‐Cy5.5 anti‐mouse CD11b; 0.5 μg/test, BioLegend, #101227) antibodies at 4°C for 30 min staining. Then the cells were washed with PBS, fixed with Fixation buffer (Fixation Buffer, BioLegend, #420801), and cell penetration was performed using 0.5% Triton X‐100. After cell resuspension with buffer containing 2% FBS in PBS, APC anti‐mouse CD206 antibody (0.5 μg/tests, BioLegend, #141707) was added and incubated for 30 min at room temperature and protected from light. Finally, the treated cell suspension was placed into a flow cytometer for detection.

### 2.11 Statistical analysis

All statistical analyses were performed using R language 4.2.1 and/or GraphPad Prism 7.0 software. Expression of KCND2 in tissues was analyzed by Wilcoxon rank sum test. Survival curves were performed using the Kaplan–Meier method. Immune cell infiltration analysis was conducted with Spearman's test. Two‐tailed *t*‐test was used for two groups analysis. *p* < 0.05 was considered to be statistically significant.

## 3 RESULTS

### 3.1 KCND2 is highly expressed in gastric cancer and correlates with the clinical features and prognosis of patients

**FIGURE 1:**
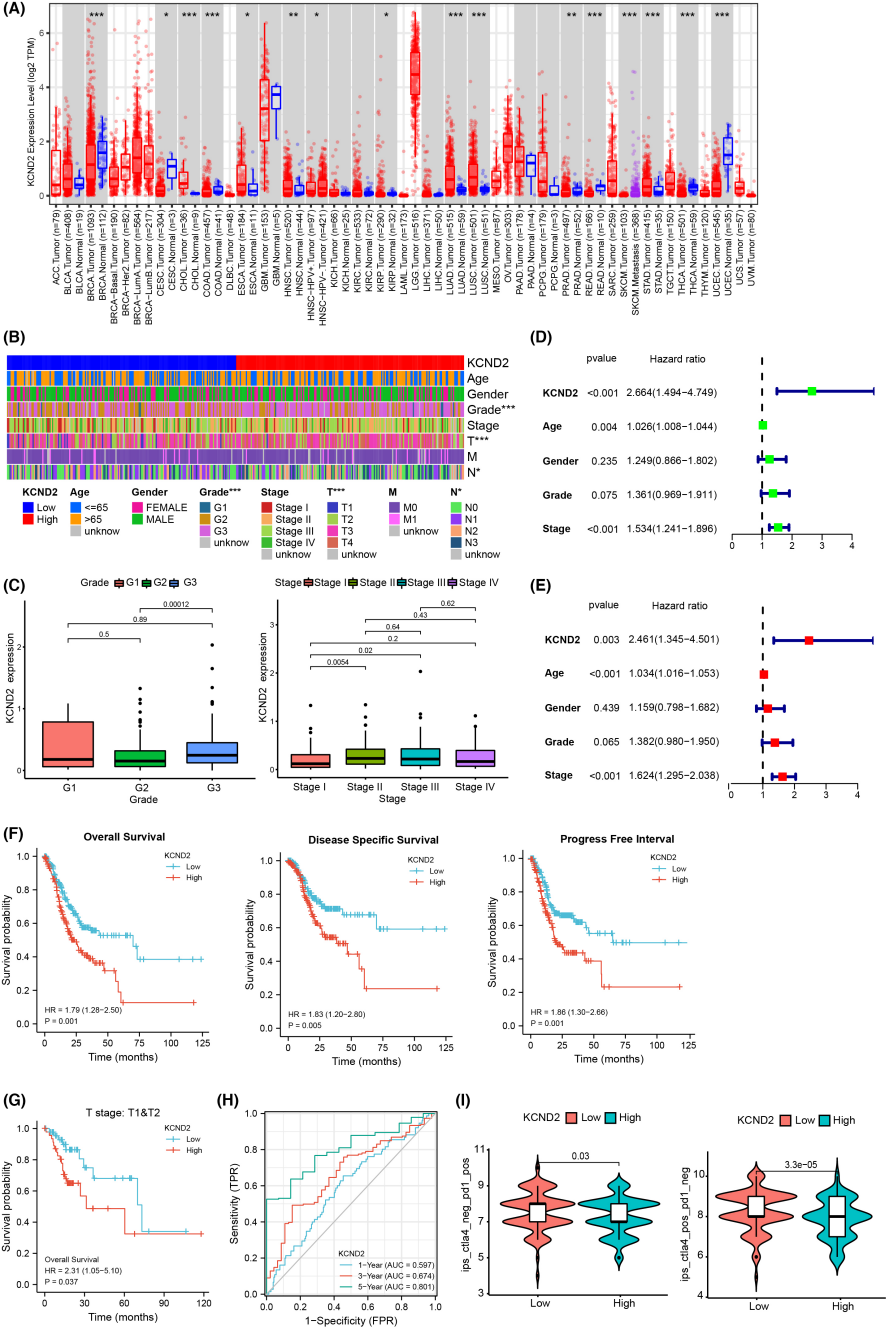
KCND2 is highly expressed in gastric cancer and correlates with the clinical features and prognosis of patients. (A) The expression levels of KCND2 in normal and different tumor tissues from TCGA database generated by TIMER2.0 database (http://timer.comp‐genomics.org/).^46^ (B) The correlation between KCND2 expression and different clinical characteristics, such as age, gender, and stages. (C) Relevance of KCND2 levels to grade (including G1, G2, and G3), TNM stages (including Stage I, Stage II, Stage III, and Stage IV). (D, E) Univariate analysis (D) or multifactorial analysis (E) of the link between clinical characteristics including mRNA levels of KCND2, age, gender, stage, and overall patient survival (OS) of gastric cancer patients from TCGA database. (F) The high levels of KCND2 exhibited worse overall survival, disease‐specific survival, and progress‐free interval in the patients with gastric cancer. (G) The correlation of KCND2 levels in different T stages (T1 and T2) patients. (H) The time ROC analysis was used for testing the AUC curve. (I) The correlation between KCND2 expression and the effectiveness of PD1 or CTLA4 immunosuppressive therapy. *p < 0.05; **p < 0.01; ***p < 0.001.

In our pursuit to understand the influence of KCND family genes on GC, we conducted a comprehensive correlation analysis using the TCGA database. This analysis encompassed all members of the KCND family, including KCND1, KCND2, and KCND3, with a specific focus on their relationship with the overall survival (OS) of GC patients. Intriguingly, among the KCND family genes examined, only KCND2 exhibited a significant effect on OS in GC (Figure S1). We next examined in all the tumors for levels of KCND2, and it was observed that KCND2 was found to be high in a variety of tumors, including gastric cancer from TCGA database (Figure 1A). Subsequently, the impact of KCND2 on the clinicopathologic characteristics and survival time of patients with gastric cancer was explored. Our data displayed that KCND2 expression levels correlated with different grades, T stages and N stages (Figure 1B), with higher grade (G3) exhibiting higher KCND2 expression than lower grade (G1); grader T stages showed increased levels of KCND2 expression (Figure 1C). Univariate and multifactorial analyses demonstrated that KCND2 was an independent predictor of prognosis in gastric cancer (Figure 1D,E). In addition, KCND2 high expression experienced worse prognosis, which included OS, disease‐specific survival (DSF) and progress free interval (PFI) in the patients with gastric cancer (Figure 1F). Additionally, an increased T stage (T2) was found to show worse overall survival than a low T stage (T1) (Figure 1G). An area under the curve (AUC) of 0.801 at 5 years was revealed by the time‐ROC curve, indicating that KCND2 expression was a good predictor of prognostic survival (Figure 1H).

Furthermore, we assessed the significance of KCND2 expression in relation to clinically relevant OS outcomes in GC samples using the Kaplan–Meier plotter (https://kmplot.com/analysis/). This valuable resource integrates data from three prominent global cancer centers located in Europe, the United States, and Australia, along with other publicly available datasets, resulting in a comprehensive analysis involving a total of 1065 GC cases.^18^ Our findings reveal the significant predictive value of KCND2 as a reliable indicator for OS in a specific subset of GC patients. Our study highlights KCND2 as a promising biomarker, particularly in well‐differentiated, intestinal type, and Stage III patients (Table 1). Notably, KCND2 expression proved to be an informative predictor of OS regardless of the patient's HER2 status, whether HER2‐negative or HER2‐positive (Table 1).

**TABLE 1:** Correlation analysis of KCND2 expression in relation to clinically informative OS in GC samples generated by Kaplan–Meier plotter.

Clinical cohorts	KCND2 expression		HR (95% CI)	*p* value
	Low (*n*)	High (*n*)		
Differentiation				
Poorly differentiated	116	49	1.22 (0.8–1.85)	0.35
Moderately differentiated	18	49	1.7 (0.71–4.07)	0.23
Well differentiated	13	19	4.95 (1.62–15.06)	0.0021**
Perforation				
No	124	45	1.5 (0.98–2.29)	0.058
Lauren classification				
Instestinal	165	155	1.87 (1.36–2.58)	9.7e‐05^****^
Diffuse	179	62	1.41 (0.98–2.03)	0.063
Mixed	9	23	3.5 (0.78–15.62)	0.081
TNM Stage				
Stage I	46	21	2.16 (0.81–5.78)	0.12
Stage II	89	51	1.46 (0.8–2.65)	0.21
Stage III	228	77	1.5 (1.1–2.04)	0.0091**
Stage IV	65	83	1.46 (0.99–2.17)	0.055
HER2 status				
HER2 (−)	329	203	1.36 (1.09–1.71)	0.0068**
HER2 (+)	221	122	1.86 (1.43–2.41)	2.9e‐06^****^
Treatment				
Surgery alone	184	196	1.55 (1.16–2.07)	0.0031**
5‐FU‐based adjuvant	108	44	1.21 (0.83–1.76)	0.32
Other adjuvant	56	20	2.06 (0.84–5.04)	0.11

*Note*: Full name of 5‐FU is fluorouracil; Only four samples had perforation status, thus the sample numbers are too low for meaningful analysis.

** *p* < 0.01.

^****^ *p* < 0.0001.

In the realm of clinically approved therapeutic targets for GC, the primary focus revolves around HER2, VEGF, and immunotherapy targets.^1^ Our study aimed to delve deeper into the potential associations between KCND2 and these pivotal target‐related genes. Our findings indicate that KCND2 demonstrates a moderate association with VEGF targets, a slight association with immunotherapeutic targets, while KCND2 might not directly impact HER2‐related pathways in GC (Table S1). Next, our analysis explored the relationship between KCND2 expression and the effectiveness of PD1 or CTLA4 immunosuppressive therapy. Surprisingly, we found that low expression of KCND2 was associated with improved efficacy of anti‐PD1 or anti‐CTLA4 treatment, suggesting that high KCND2 expression may impede the effectiveness of immunosuppressive therapy (Figure 1I).

### 3.2 KCND2 promotes the growth of gastric cancer cells in vitro

**FIGURE 2:**
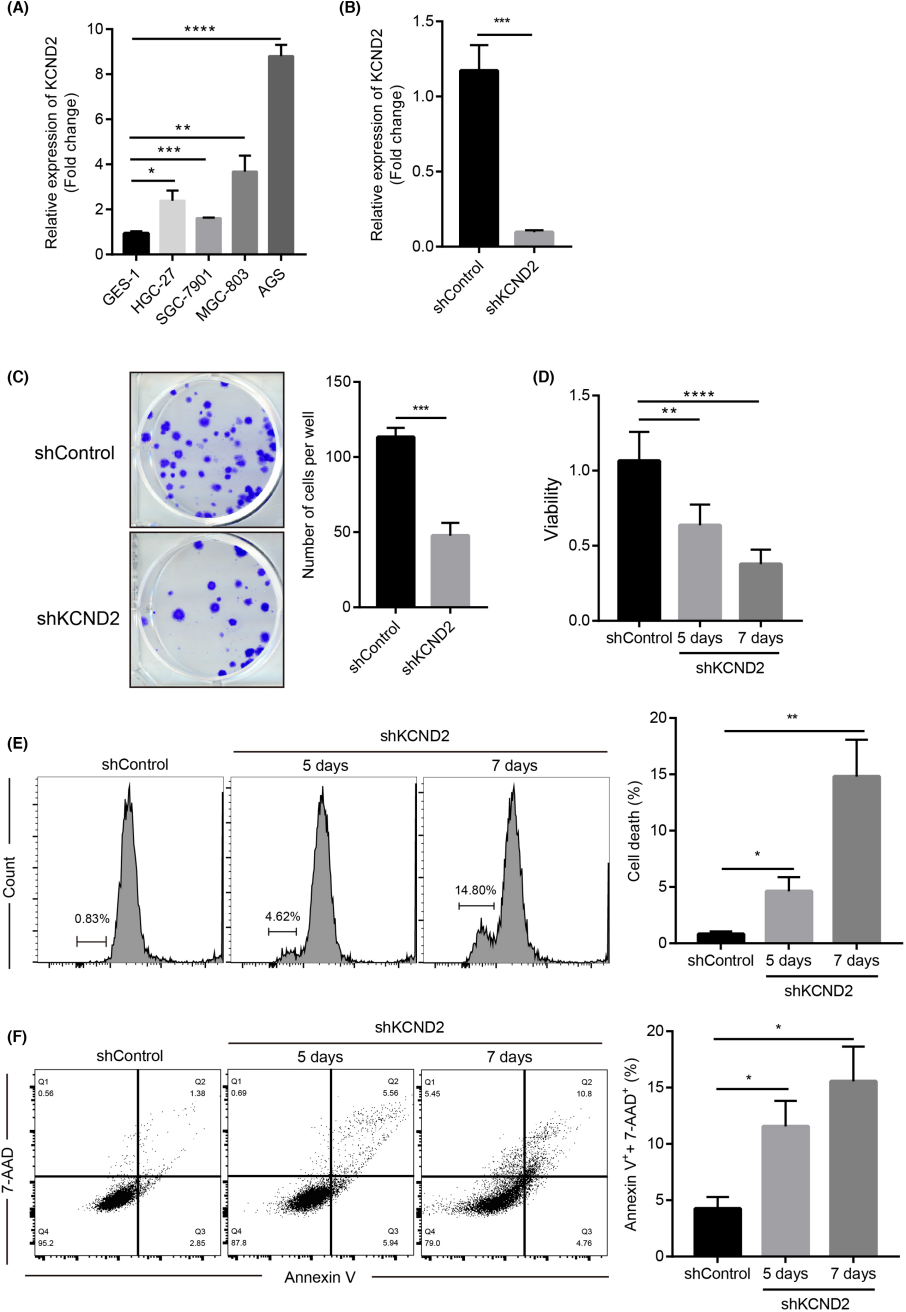
KCND2 promotes the growth of gastric cancer cells in vitro. (A) The mRNA levels of KCND2 in GES‐1, HGC‐27, SGC‐7901, MGC‐803, and AGS were detected by qRT‐PCR. (B) Knockdown efficiency of KCND2 was utilized with qRT‐PCR assay. (C) Control or KCND2 knockdown AGS cells were transfected and seeded in 6‐well plates and observed with clonal formation experiments at Day 14. (D) Control or KCND2 knockdown AGS cells were transfected and seeded in 96‐well plates for detecting the viability by CCK8 assay at Day 5 or Day 7. (E, F) AGS cells were transfected with control or KCND2 knockdown for 5 or 7 days and tested with cell cycle (E) or Annexin V and 7‐AAD staining by flow cytometry assays. *p < 0.05; **p < 0.01; ***p < 0.001; ****p < 0.0001.

With a view to further verifying the capabilities of KCND2 on gastric cancer cells, we first examined the levels of KCND2 in GES‐1 cells (human gastric epithelial cells) and SGC‐7901, HGC‐27, AGS and MGC‐803 cells (gastric cancer cells) by qRT‐PCR. The mRNA levels of KCND2 were significantly enhanced in s all gastric cancer cell lines compared to GES‐1 cells, with the highest expression levels in AGS cell lines (Figure 2A). Therefore, we knocked down the levels of KCND2 in AGS cells and confirmed its efficiency by qPT‐PCR, which was validated and revealed that the mRNA levels of KCND2 were greatly declined in both the KCND2 knockdown and control groups (Figure 2B). To detect the effect of KCND2 on AGS cell growth, clone formation experiments showed that knocking down KCND2 led to reducing the proliferation of gastric cancer cells (Figure 2C). Similarly, CCK8 proliferation assay displayed that the cell viability of AGS cells in the KCND2 knockdown group was markedly decreased on Day 5 and Day 7, and the cell viability decreased more obviously on Day 7 (Figure 2D). Further, cell cycle results revealed that the death rate of AGS cells was considerably higher in shKCND2 compared to controls with a time‐dependent manner (Figure 2E). Similarly, AGS cells treated with shKCND2 for 5 and 7 days showed a higher rate of positive Annexin V and 7‐AAD cells by flow cytometry compared to the control group (Figure 2F).

### 3.3 KCND2 is associated with M2 macrophages

**FIGURE 3:**
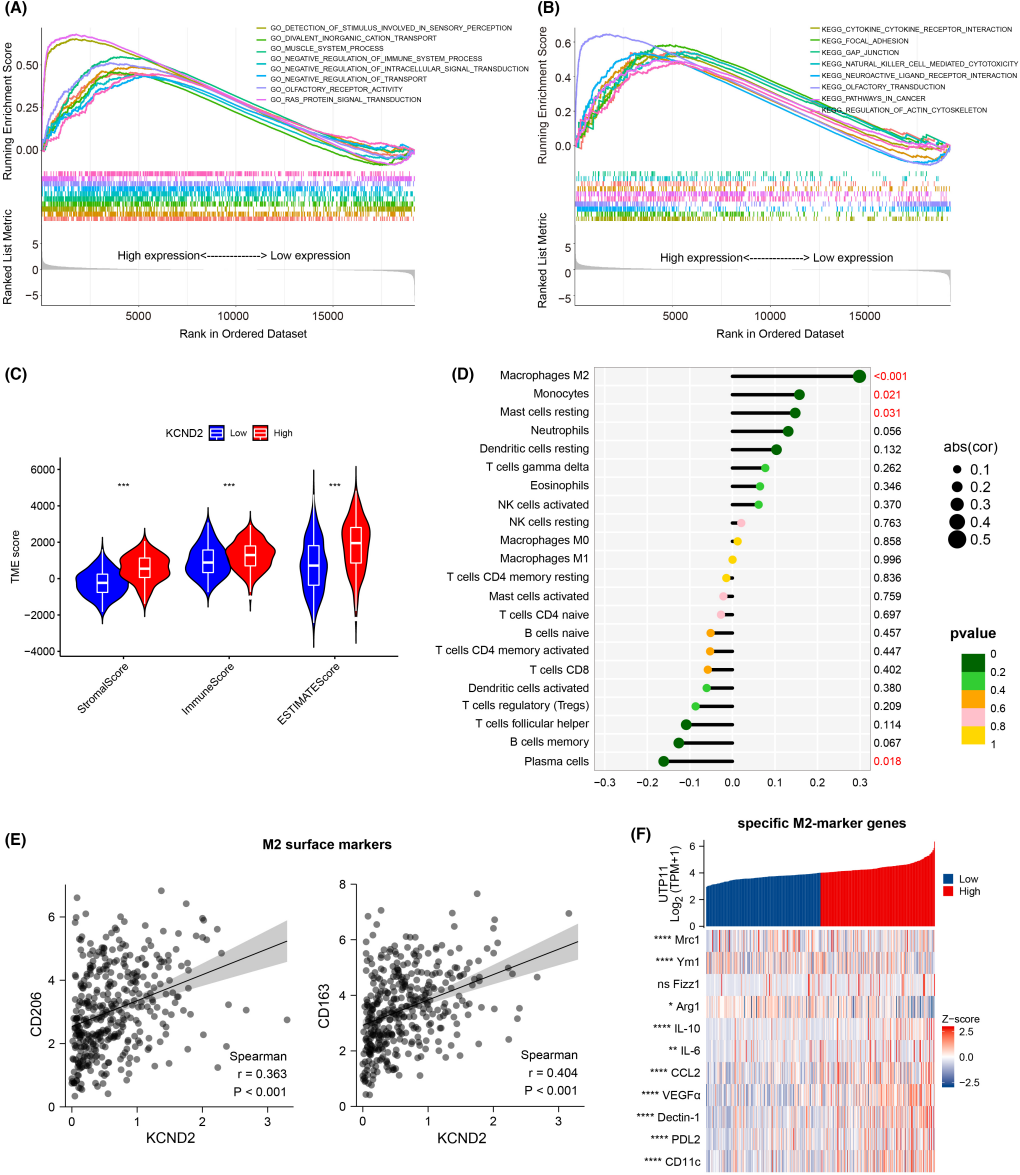
KCND2 is associated with M2 macrophages. (A, B) GO (A) and KEEG (B) analysis were used to inspect the relevant functions and signaling pathways of KCND2 from TCGA database. (C) The correlation of KCND2 levels with stromal, immune in the TCGA database, and the high levels of KCND2 were positively correlated with both stromal and immune scores. (D) We analyzed the correlation of KCND2 expression with various immune cell types, and observed that it was positively associated with M2 macrophages, monocytes, and resting mast cells, while negatively correlated with plasma cells. (E) KCND2 was strongly linked to the M2 macrophage surface markers CD206 and CD163. (F) KCND2 expression was positively correlated with specific M2 macrophage marker genes, including Mrc1, Ym1, Arg1, and IL‐10. *p < 0.05; **p < 0.01; ***p < 0.001; ****p < 0.0001.

To further explore the mechanism of how KCND2 promotes the growth of gastric cancer, we undertook GO‐KEEG functional as well as enrichment pathway analysis, and our data showed that KCND2 expression was associated with immune system process, cytokine‐cytokine receptor interaction, nature killer cell mediate cytotoxicity (Figure 3A,B), suggesting that KCND2 might regulate the immune system. Therefore, we explored the relevance of KCND2 levels to stroma and immunity in the TCGA database. The findings suggested that the high expression levels of KCND2 were positively correlated to both stromal and immune scores, that is, the greater the expression of KCND2, the higher the stromal and immune scores (Figure 3C). Further, it was also analyzed the associations of KCND2 expression with a variety of different inflammatory immune cells. The outcomes demonstrated that high KCND2 expression was favorably linked to M2 macrophages, monocytes, mast cells resting, and negatively related to plasma cells, with the most associated with M2 macrophages (Figure 3D). In addition, KCND2 was found to be strongly associated with the M2 macrophage surface markers CD206 and CD163 (Figure 3E). Furthermore, KCND2 expression was positively correlated with several specific M2 macrophage marker genes, such as Mrc1, Ym1, Arg1, and IL‐10 (Figure 3F). These results provide further evidence of KCND2's involvement in regulating M2 macrophage activity and immune function.

### 3.4 KCND2 promotes gastric cancer cell proliferation via activation of NF‐κB pathway

**FIGURE 4:**
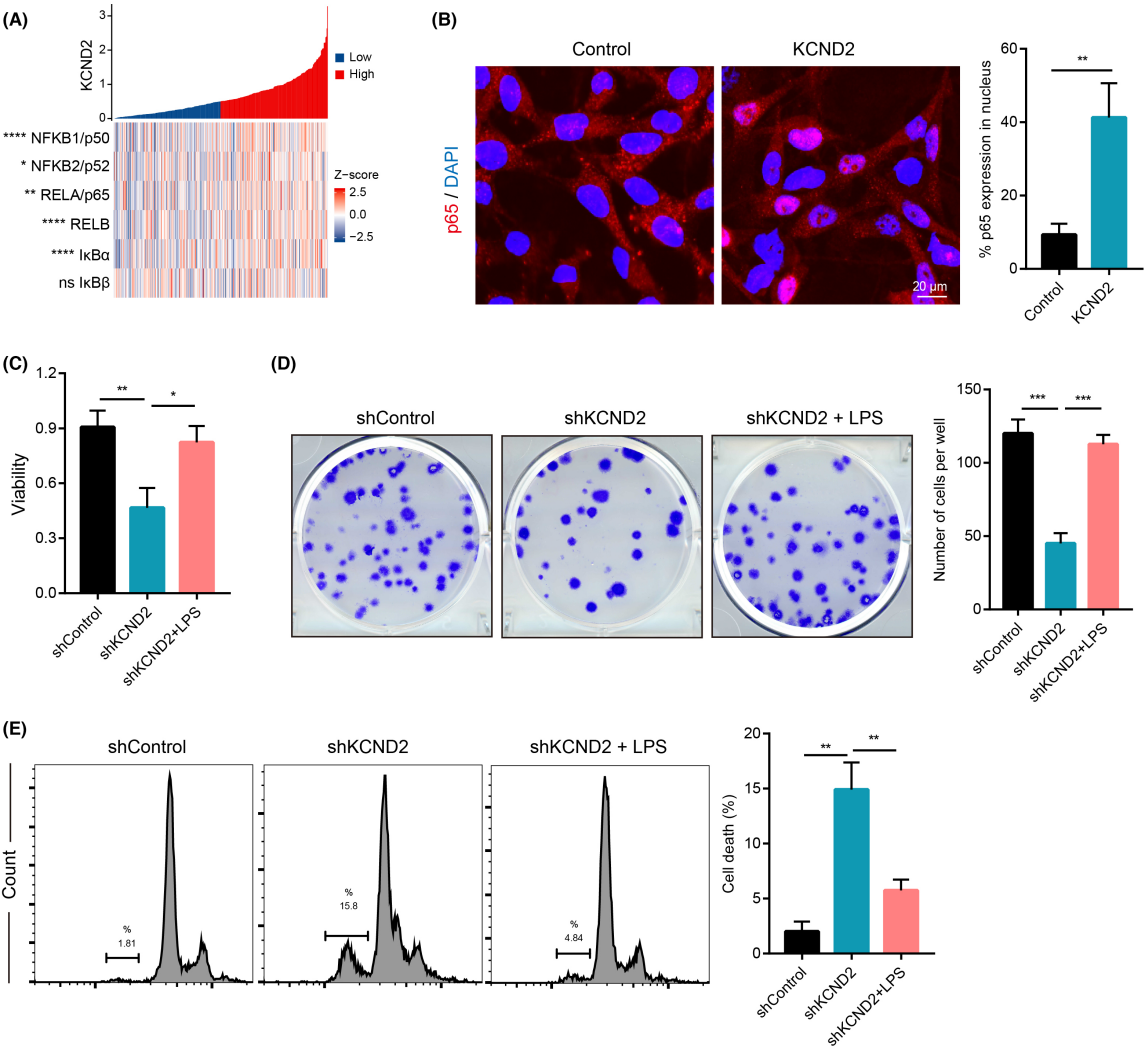
KCND2 promotes gastric cancer cell proliferation via activation of NF‐κB pathway. (A) KCND2 was positively associated with several NF‐κB pathway‐related factors, including p50, p52, p65, RELB, and IκBɑ from TCGA database. (B) The protein expression of NF‐κB p65 was detected in control or KCND2 overexpressing cells using immunofluorescence assay. We observed an increase in the nuclear translocation of NF‐κB p65 in high levels of KCND2 cells compared to control. (C) Control or KCND2 knockdown AGS cells were treated with PBS or LPS (100 ng/mL) for 7 days, and cell viability was measured by CCK8. (D) Treatment with PBS or LPS (100 ng/mL) for 7 days in control or KCND2 knockdown cells and the clone formation experiments were performed. (E) Control or KCND2 knockdown AGS cells were treated with PBS or LPS (100 ng/mL) for 7 days, and then detection with cell cycle. *p < 0.05; **p < 0.01; ***p < 0.001.

NF‐κB is an extremely common transcription element which is present in virtually every animal cell and participates in a variety of cytological responses to external stimuli, like cytokines, radiation, viruses, and heavy metals.^19^ As a key regulator of cellular immune responses and tumor progression,^20^, ^21^ NF‐κB is an important target for investigation in cancer research. Given its importance, we sought to determine whether KCND2 facilitates the development of gastric cancer via activating the NF‐κB pathway. To begin with, we examined the correlation of KCND2 levels with relevant factors of the NF‐κB using data from the TCGA database. Interestingly, we found that KCND2 was positively associated with multiple NF‐κB‐related parameters, such as p50, p52, p65, RELB, and IκBɑ (Figure 4A).

NF‐κB pathway is activated by the phosphorylation and degradation of its inhibitory subunit, IκB, which is subsequently translocated by NF‐κB subunits, such as p65, from the cytoplasm to the nucleus.^19^ Therefore, we next overexpressed KCND2 in gastric cancer cells to verify the visualization of nuclear translocations of NF‐κB p65, which is a hallmark of NF‐κB activation. Our findings revealed that high KCND2 expression facilitated the nuclear translocation of p65, indicating the NF‐κB pathway was activated (Figure 4B). Lipopolysaccharide (LPS) activates NF‐κB, leading to the subsequent transcription and expression of inflammatory factors, and for this reason, LPS is commonly used as an NF‐κB activator.^22^ Aiming to understand better the contribution of KCND2 in promoting gastric cancer cell proliferation through NF‐κB activation, we knocked down KCND2 expression and treated cells with lipopolysaccharide (LPS), a commonly used activator of the NF‐κB pathway. KCND2 knockdown suppressed cell proliferation, whereas LPS treatment reversed this effect with CCK8 and clone formation assays (Figure 4C,D). Similarly, KCND2 knockdown enhanced the rates of subG1 cells, a hallmark of apoptosis, while LPS alleviated this effect. These results suggest that KCND2 contributes to gastric cancer cell proliferation via activating NF‐κB (Figure 4E). The above results concluded that KCND2 accelerates the cell proliferation of gastric cancer cells by activating NF‐κB pathway.

### 3.5 KCND2 boosts the growth of gastric cancer by activating the NF‐κB pathway in vivo

**FIGURE 5:**
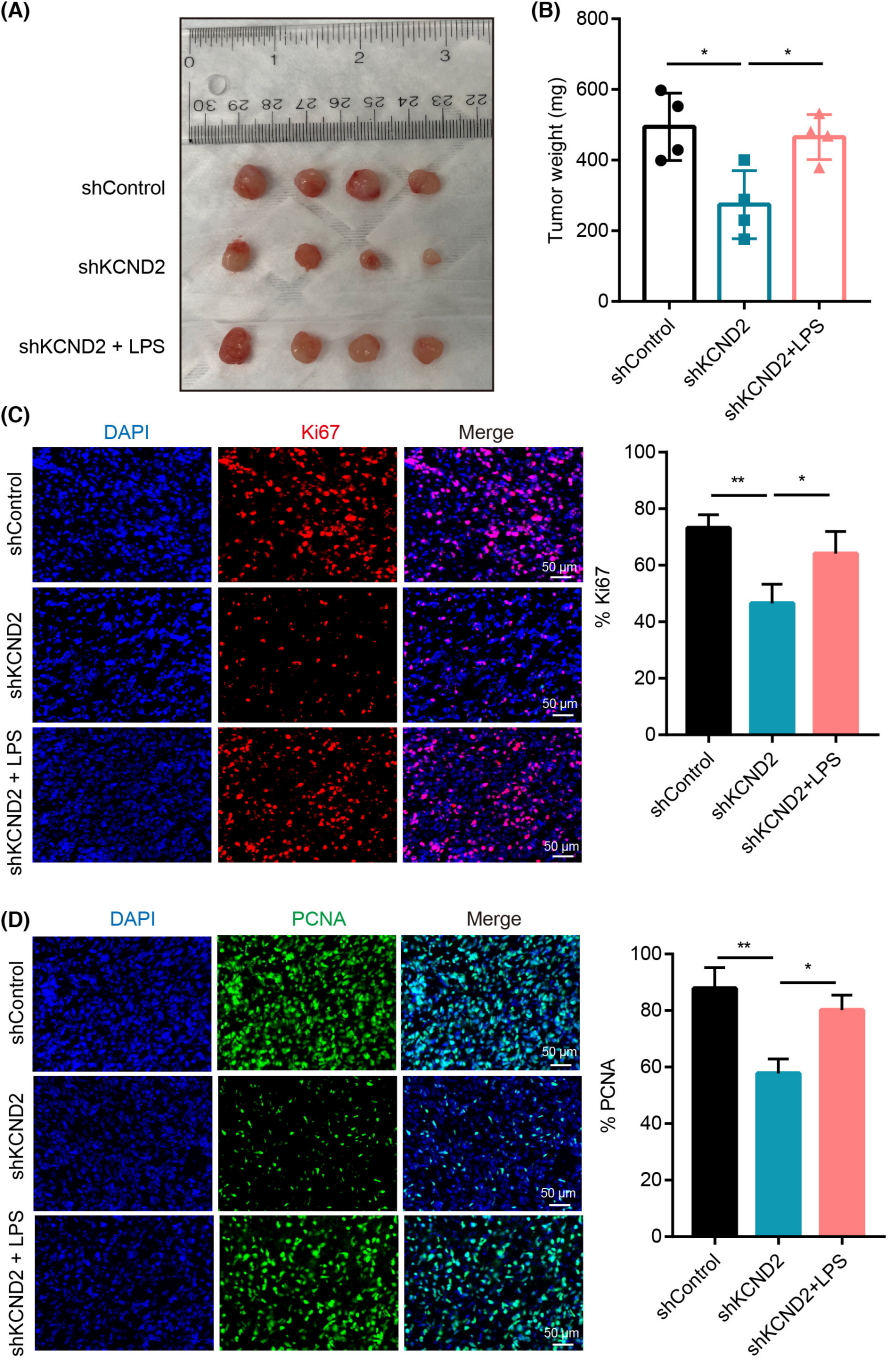
KCND2 boosts the growth of gastric cancer by activating the NF‐κB pathway in vivo. (A, B) We subcutaneously injected control or KCND2 knockdown murine‐derived gastric cancer cells (MFC) into mice and administered PBS or LPS once the tumors grew large enough to be visualized. The data showed that knockdown of KCND2 prevented tumor growth compared with controls, while LPS treatment reversed the shrinkage of tumors caused by KCND2 knockdown. (C, D) KCND2 knockdown reduced the protein expression levels of the proliferation markers Ki67 (C) and PCNA (D), while LPS treatment relieved this effect. *p < 0.05; **p < 0.01.

To learn more about the impact of KCND2 on gastric cancer in animal experiments and further understand its potential mechanisms, murine‐derived gastric cancer cells MFC with control or KCND2 knockdown were subcutaneously injected into 615 mice, followed by intraperitoneal injection of DMSO or LPS, when the tumors grew large enough to be visualized. Consistent with the results from cellular studies, knockdown of KCND2 prevented tumor growth compared with controls, while activating of the NF‐κB by LPS reversed the shrinkage among tumors due to KCND2 knockdown (Figure 5A,B). Furthermore, compared to the control group, KCND2 knockdown was able to diminish the protein expression levels of the proliferation indicators Ki67 and proliferating cell nuclear antigen (PCNA), while LPS can relieve the effects of the reduction in expression levels resulting in KCND2 knockdown (Figure 5C,D). Thus, these findings demonstrated that KCND2‐enhanced gastric cancer growth was achieved through NF‐κB pathway activation.

### 3.6 KCND2 promotes M2 macrophage polarization through the activation of NF‐κB pathway in vivo

**FIGURE 6:**
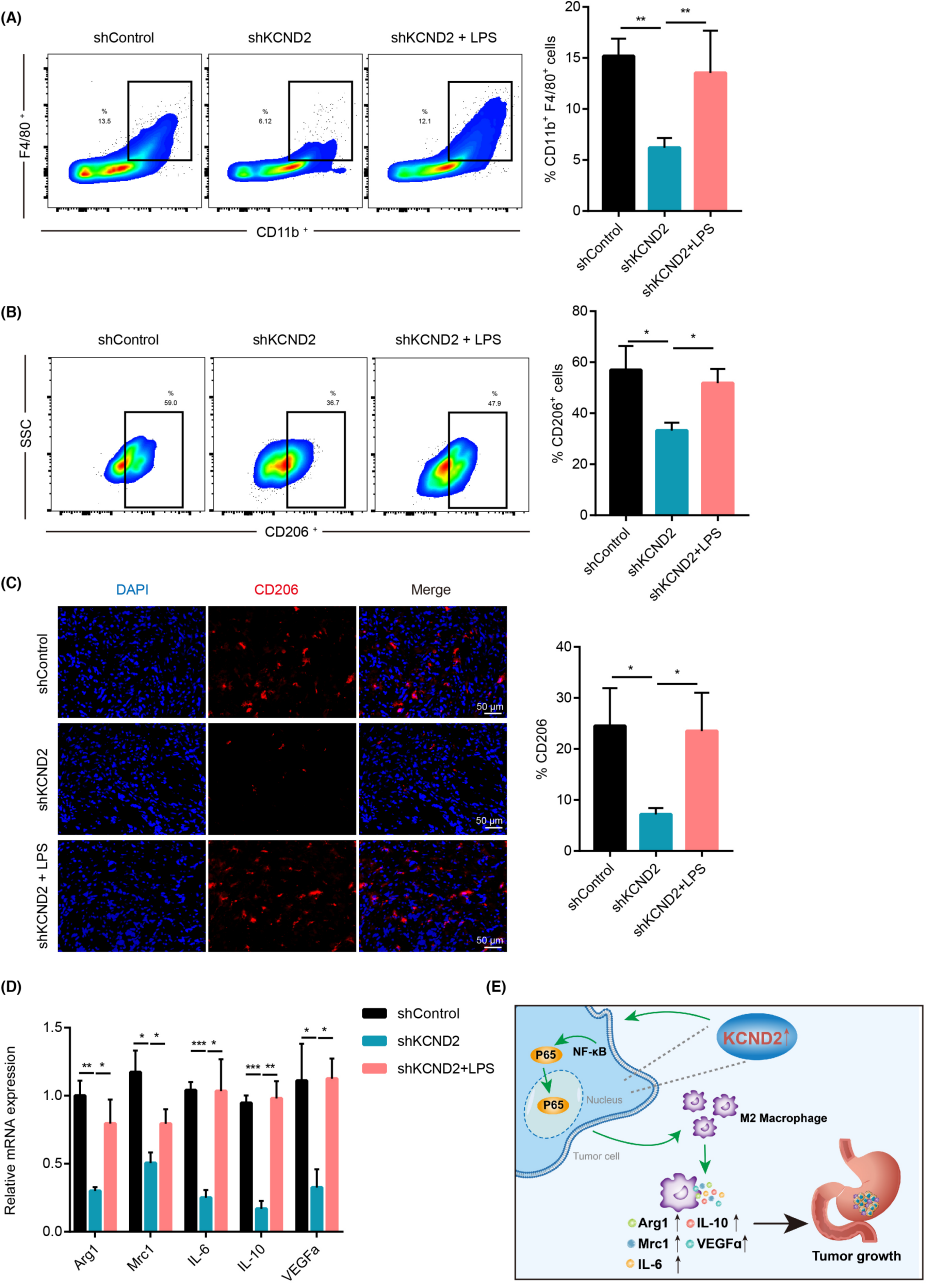
KCND2 promotes M2 macrophage polarization through the activation of NF‐κB pathway in vivo. (A, B) Flow cytometry results showed that KCND2 knockdown reduced CD11b^+^F4/80^+^ macrophage infiltration (A) and the CD11b^+^F4/80^+^CD206^+^ (B) ratio compared to the control group. On the other hand, the activation of the NF‐κB pathway by LPS increased the ratio of CD11b^+^F4/80^+^ cells (A) and CD11b^+^F4/80^+^CD206^+^ cells (B) compared to the KCND2 knockdown groups. (C) KCND2 knockdown inhibited the protein expression levels of CD206, while LPS reversed the decrease in CD206 expression caused by KCND2 knockdown, as verified by immunofluorescence assay. (D) KCND2 knockdown also suppressed the secretion of factors such as Arg1, Mrc1, IL‐6, IL‐10, and VEGF compared to the control group, whereas LPS increased the expression of these secretory factors resulting from KCND2 knockdown. (E) Schematic diagram of KCND2 promotes gastric cancer. *p < 0.05; **p < 0.01; ***p < 0.001.

The above findings suggest a robust positive link between KCND2 and M2 macrophages, as well as their surface antigens and secreted factors (as shown in Figure 3). Therefore, we next performed animal experiments to demonstrate the relationship between KCND2 and macrophage infiltration in vivo. Flow cytometry results exhibited that KCND2 knockdown reduced CD11b^+^F4/80^+^ macrophage infiltration and CD11b^+^F4/80^+^CD206^+^ ratio compared to control group (Figure 6A,B). Moreover, LPS activation toward NF‐κB by LPS increased the proportion of CD11b^+^F4/80^+^ cells and CD11b^+^F4/80^+^CD206^+^ cells compared to KCND2 knocking down groups (Figure 6A,B). Accordingly, KCND2 knockdown suppressed the protein expression levels of CD206, while LPS reversed the decrease in CD206 expression caused by KCND2 knockdown via immunofluorescence assay. In addition, KCND2 knockdown inhibited the M2 macrophages secretion of factors such as Arg1, Mrc1, IL‐6, IL‐10, and VEGF compared to controls, whereas LPS added the expression levels of these secretory factors resulting from KCND2 knockdown (Figure 6D). In conclusion, KCND2 contributes to the growth of gastric cancer by activating the NF‐κB through promoting the infiltration of M2 macrophages (Figure 6E).

## 4 DISCUSSION

During latest years, a growing amount of findings have been documented that dysregulated Kv channels are important in the pathological advancement of a wide range of cancers, such as gastric,^23^ breast,^24^ and colorectal cancers.^25^, ^26^ KCND family members are involved in the biology of different cancers. Knockdown of KCND1 inhibited the proliferation of gastric cancer cells.^11^ Upregulation of KCND1 promoted tumorigenic effects in human breast epithelial cells, while repression of KCND1 expression suppressed the proliferative effects of breast cancer cells.^10^ Recent studies have shown that large T antigen promotes KCND3 expression, whereas inhibiting KCND3 expression could induce apoptosis and necrosis. Park JH et al. reported that high expression of KCND2 favored the tumor stem cell properties of neuroblastoma cells.^12^ KCND2 is also a pivotal gene implicated in the initiation and pathogenesis of bladder cancer.^13^ KCND2 appears to be overexpressed in lung adenocarcinoma (AD) and its reduction could prevent the proliferation, invasion and migration of AD cells.^27^ Further, high expression of KCND2 was associated with prognostic survival of gastric cancer^28^; however, it is unknown whether KCND2 acts in the growth process of gastric cancer. In similarity with previous publications, our article illustrated that KCND2 in gastric cancer patients had been shown to be highly expressed and linked to poor clinical features and prognosis. Importantly, our study revealed that KCND2 was able to initiate the development of gastric cancer cells at both the cellular and animal levels, suggesting that KCND2 is critical for the formation and progression of gastric cancer. Strikingly, our results also indicated that low KCND2 expression was associated with a more favorable response to anti‐PD1 or anti‐CTLA4 treatment, suggesting that elevated KCND2 expression might have a detrimental effect on the effectiveness of immunosuppressive therapy. Further research is necessary to unravel the underlying mechanisms by which KCND2 influences the response to immunosuppressive therapy. These findings hold the potential to facilitate the development of personalized treatment approaches and the identification of potential biomarkers to predict patient responsiveness to immunotherapy in the context of GC.

There are several reports identifying KCND family genes that are potentially closely involved in cancer initiation and development, however, its mechanism of promoting cancer advancement has been studied to a limited extent. The current mechanisms by which KCND family genes contribute to neoplastic growth are as follows. Knockdown of KCND1 induced cell cycle G1‐S blockade and thus repressed the formation of gastric cancer cells.^11^ The overexpression of microtubule binding protein tau resulted in a decrease in the mRNA expression levels of KCND1, thereby suppressing the growth of murine neuroblastoma N2A cells.^29^ Large conductance Ca (2+)‐activated KCND2 overexpression increased the percentage of CD133^+^ subpopulation in neuroblastoma SH‐SY5Y cells and contributed to the increase of cancer stem cells (CSCs).^12^ The downregulation of EMT‐related genes such as SNAI1, MMP2, and CDH2 by KCND2 encourages the aggressiveness of AD cells.^27^

By profiling the TCGA database, we found that KCND2 was involved in tumor‐associated pathways and immune processes through GO and KEGG enrichment. Interestingly, KCND2 was found to be in positive correspondence with stromal cell and immune cell scores, suggesting that KCND2 appeared to be able to regulate the tumor microenvironment (TME). TME is defined as the non‐cancerous component that exists in the neoplasm and cancer cells portray the microenvironment through various cellular molecules, chemokines produced and released by cancer cells.^30^, ^31^ Among them, immune cells serve as a vital component in TME.^31^ Tumor‐associated macrophages (TAMs) act in the heart of the network of immunosuppressor and cytokines, which serve essential needs in neoplastic immune evasion.^32^, ^33^ TAMs are functionally heterogeneous and are predominantly categorized into two key subpopulations, M1 and M2 macrophages.^32^ M1 macrophages are considered to provide the primary line of protection against microbial infection, as well as having a strong antigen‐presenting capacity and inducing a robust Th1 response.^34^ In contrast, M2 macrophages hold essential positions in limiting immune responses and eliciting angiogenesis and induction of tissue repair; therefore, their presence is implicated in prospective tumorigenic activity, whereas the existence of M1 TAMs favors antitumorigenic activity.^32^, ^34^ In this study, KCND2 expression was positively correlated with M2 macrophage from TCGA database, and KCND2 could promote the infiltration of M2 macrophages of gastric cancer in vivo, which may be responsible for facilitating the growth of gastric cancer.

Participation of TAM in immune evasion is extensively reported in the literatures, and the following mechanisms are currently recognized. TAM alters TAM polarization by regulating helper T cells (Th), specifically, Th1 cells contribute to M1 polarization by secreting IFN‐γ, in contrast with Th2 cells that produce IL‐4 and IL‐10 to boost the generation of M2 macrophages.^35^ It is also known that cancer‐associated fibroblasts (CAF) secrete M‐CSF, IL‐6 and MCP‐1 factors, which facilitate macrophage inflammation and polarization, while M2 produce TGF‐β, stimulating the conversion of endothelial cells to mesenchymal cells and increasing the responsiveness of CAF, thus strengthening the aggressiveness of cancer cells.^36^ M1 macrophages can generate anti‐inflammatory molecules such as TNF‐α, IL‐23, and iNOS, by triggering inflammatory responses and exerting anti‐tumor effects. In contrast, M2 macrophages are capable of secreting cytokines that facilitate immunosuppressive and tumor‐promoting activities, such as TGF‐β, Arg‐1, Mrc1, IL‐10, etc.^37^, ^38^ Here, we showed that KCND2 knockdown inhibited the M2 macrophages secretion of factors such as Arg1, Mrc1, IL‐6, IL‐10, and VEGF compared to controls in vivo, suggesting that KCND2 might potentially promote the growth of gastric cancer through activation of M2 macrophages.

Growing investigations have identified the importance of the NF‐κB in cancer‐related immune responses and neoplastic malignant development.^39^, ^40^ Hagemann T et al. discovered that TAMs were polarized to an immunosuppressive phenotype via IL‐1R and MyD88, which required I kappa B kinase β‐mediated NF‐κB activation to achieve this process.^41^ It was shown that NF‐κB signaling targeting in TAMs was capable at inducing tumor‐killing activity in TAMs and activating anti‐tumor activity via recruitment of IL‐12‐dependent NK cells to promote tumor regression in vivo.^41^, ^42^ Our finding revealed that the ability of KCND2 to promote infiltration of M2 macrophages was achieved through NF‐κB activation in animal experiment. However, how KCND2 regulates the activation of the NF‐κB by M2 macrophages remains unclear.

Signal transducer and activator of transcription (STAT) proteins hold central function in the tumor immune response in TME, and STAT3 promotes a pro‐cancer inflammatory response via NF‐κB, implying that STAT3 links inflammation and cancer.^21^ The STAT3/NF‐κB pathway is widely documented to stimulate the polarization of M2 macrophages, hence promoting cancer progression.^43^, ^44^, ^45^ It is well documented that KCND2 upregulates STAT3 in ischemic stroke; therefore, there is speculation whether KCND2 can regulate STAT3 in response to NF‐κB pathway activation, which we will confirm with more follow‐up studies.

In conclusion, our article illustrated that KCND2 was upregulated in gastric cancer patients and was implicated in adverse clinical features and clinical prognosis. Furthermore, knocking down KCND2 in cellular and animal experiments resulted in the ability to inhibit gastric cancer cell viability and growth. Finally, by mechanism, KCND2 was shown to lead to the infiltration of M2 macrophages through activation of NF‐κB to promote the advancement of gastric cancer eventually. Overall, this research provides promising insights into the involvement of KCND2 in the development of gastric cancer and suggests that targeting KCND2 may serve a potential therapeutic strategy for the treating gastric cancer.

## AUTHOR CONTRIBUTIONS

**Hongying Zhou:** Data curation (lead); formal analysis (lead); funding acquisition (lead); investigation (lead); methodology (lead); software (lead); validation (lead); writing – original draft (lead); writing – review and editing (lead). **Dan Su:** Data curation (equal); formal analysis (equal); methodology (equal); validation (equal); writing – review and editing (equal). **Yun Chen:** Data curation (equal); methodology (equal); validation (supporting); writing – review and editing (supporting). **Yiwen Zhang:** Writing – review and editing (supporting). **Ping Huang:** Data curation (equal); project administration (lead); supervision (lead); validation (equal); writing – review and editing (lead).

## FUNDING INFORMATION

This study was supported by the Zhejiang medical and health research project (No. 2020KY407).

## CONFLICT OF INTEREST STATEMENT

These authors have no conflict of interest to declare.

## ETHICS STATEMENT

Animal experiments were approved by the Animal Ethics Committee of Zhejiang Provincial People's Hospital (Ethics No. IACUC‐20230306‐03).

## Supporting information


Figure S1

Table S1

## Data Availability

The datasets used and/or analyzed during the current study are available from the corresponding authors upon reasonable request.
