# Supplementary figures and images for: KCND2: A prognostic biomarker and regulator of immune function in gastric cancer
Source: Cancer Med. 2023 Jun 22;12(15):16279–94. doi: 10.1002/cam4.6236 (PMC10469724; doi:10.1002/cam4.6236)

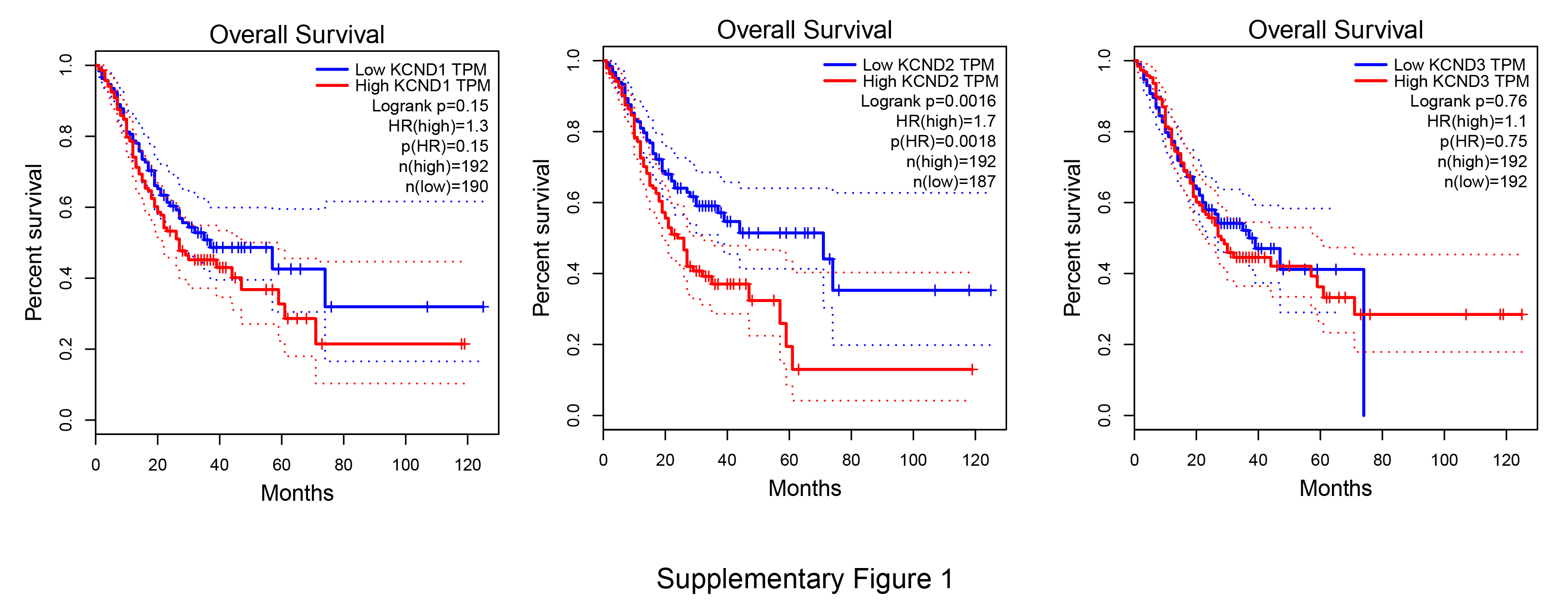

Supplement: Supplementary file 1 — Figure S1 [file CAM4-12-16279-s001.jpg]
