# Supplementary material for: KCND2: A prognostic biomarker and regulator of immune function in gastric cancer
Source: Cancer Med. 2023 Jun 22;12(15):16279–94. doi: 10.1002/cam4.6236 (PMC10469724; doi:10.1002/cam4.6236)
Supplement: Supplementary file 2 — Table S1 [file CAM4-12-16279-s002.docx]

Supplementary Table 1: Correlation of KCND2 expression with clinically approved targeted drug targets (Her2, VEGF, Immunotherapy) for GC Treatment in the TCGA database.

| **Genes** | **Correlation coefficient** | **P value** |
| --- | --- | --- |
| ERBB2 (HER2) | -0.12 | 0.0144 |
| VEGFA | -0.098 | 0.0459 |
| VEGFB | 0.391 | 1.3e-16 |
| VEGFC | 0.633 | 6.71e-48 |
| FLT1 (VEGFR-1) | 0.346 | 4.37e-13 |
| KDR (VEGFR-2) | 0.285 | 3.59e-09 |
| FLT4 (VEGFR-3) | 0.363 | 2.4e-14 |
| CD274 (PD-L1) | 0.214 | 1.04e-05 |
| PDCD1 (PD1) | 0.115 | 0.019 |
| CTLA4 | 0.133 | 0.00674 |
